# Functionalized Selenium Nanotherapeutics Synergizes With Zoledronic Acid to Suppress Prostate Cancer Cell Growth Through Induction of Mitochondria-Mediated Apoptosis and Cell Cycle S Phase Arrest

**DOI:** 10.3389/fonc.2021.685784

**Published:** 2021-06-08

**Authors:** Yulin An, Jianfu Zhao

**Affiliations:** Research Center of Cancer Diagnosis and Therapy, Department of Oncology, The First Affiliated Hospital, Jinan University, Guangzhou, China

**Keywords:** selenium, nanomedicine, zoledronic acid, prostate cancer, apoptosis

## Abstract

The use of established drugs in new therapeutic applications has great potential for the treatment of cancers. Nanomedicine has the advantages of efficient cellular uptake and specific cell targeting. In this study, we investigate using lentinan-functionalized selenium nanoparticles (LET-SeNPs) for the treatment of prostate cancer (PCa). We used assays to demonstrate that a combination of LET-SeNPs and zoledronic acid (ZOL) can reduce PCa cell viability *in vitro*. Stability and hemocompatibility assays were used to determine the safety of the combination of LET-SeNPs and ZOL. The localization of LET-SeNPs was confirmed using fluorescence microscopy. JC-1 was used to measure the mitochondrial membrane potential, while the cellular uptake, cell cycle and apoptosis were evaluated by flow cytometry. Finally, cell migration and invasion assays were used to evaluate the effects of the combination treatment on cell migration and invasion. Under optimized conditions, we found that LET-SeNPs has good stability. The combination of LET-SeNPs and ZOL can effectively inhibit metastatic PCa cells in a concentration-dependent manner, as evidenced by cytotoxicity testing, flow cytometric analysis, and mitochondria functional test. The enhanced anti-cancer effect of LET-SeNPs and ZOL may be related to the regulation of BCL2 family proteins that could result in the release of cytochrome C from the inner membranes of mitochondria into the cytosol, accompanied by induction of cell cycle arrest at the S phase, leading to irreversible DNA damage and killing of PCa cells. Collectively, the results of this study suggest that the combination of SeNPs and ZOL can successfully inhibit the growth of PCa cells.

## Introduction

Prostate cancer (PCa) is one of the leading causes of cancer-related death among men worldwide, with an incidence and mortality of approximately 11% and 2.5%, respectively (1–3). The high morbidity and mortality of PCa have aroused great concern among scientists. PCa is common in middle-aged and elderly patients with urinary tract obstruction. In addition, disease relapse with metastasis is common. However, diagnosis of PCa is challenging in the vast majority of patients because of the insidious symptoms in the early stage of disease. The cases of advanced stage of cancer have lost the opportunity for radical treatment at the time of diagnosis (4, 5). According to the recommendations of National Comprehensive Cancer Network guideline, endocrine therapy alone or in combination with radiotherapy have been used in clinical practice for the treatment of PCa with T3–T4 stage in the TNM classification. Such treatment can easily lead to castration-resistant prostate cancer (CRPC) (6, 7). Based on the characteristics of high degree of malignancy, short median survival time, high mortality, and insensitivity to a variety of chemotherapeutic drugs, clinicians use endocrine therapy combined with chemotherapy, targeted therapy, or immunotherapy for patients with distant bone metastases supplemented with local treatment and supportive therapy (e.g., nutrition to enhance immunity, pain relief, et al) (8). The goal in the treatment of PCa is to improve the quality of life and prognosis of patients with advanced PCa. The development of a more effective treatment strategy against PCa is urgently warranted.

In recent years, zoledronic acid (ZOL), a third-generation nitrogen-containing bisphosphate drug, has been used in the treatment of Paget’s disease and osteoporosis (9–11). Clinical studies have confirmed the clinical efficacy of this drug in relieving bone metastasis of malignant tumors (12, 13). Numerous studies have shown that ZOL can delay bone metastasis of PCa, increase the number and activity of osteoblasts, and effectively inhibit the proliferation and activity of osteoclasts (14). Moreover, it can reduce the risk of osteoporosis (which reduced the risk of osteoporosis by 70% during a 3-year observation period), pathological fracture, and hypercalcemia (15, 16). It is generally accepted that ZOL inhibits the growth of tumor cells by inducing the isopentenylation of cellular proteins through the mevaleric acid pathway. This effect has been confirmed in *in vitro* experiments in osteosarcoma and breast cancer. However, the therapeutic effect and potential mechanism of ZOL in the treatment of primary cancer with high risk for bone metastasis or PCa with bone metastasis warrant further investigation. In addition, ZOL is used in the treatment of bone metastatic cancer and common diseases, such as osteoporosis (17). Adjusting the annual cycle to a monthly cycle increases the occurrence of common side effects of drugs, such as headache, dizziness, bone pain, gastrointestinal reactions, nephrotoxicity, et al. Also, there is insufficient evidence to support continuous use of the drug for more than three years (18). Hence, the current clinical application status limits the use of ZOL.

Selenium (Se) is one of the essential and unique trace elements in the human body. It is mainly present in the form of selenocysteine in protein and participates in immunity, reproduction, and other important functions of the body (19, 20). Selenoprotein is present in the 21st amino acid selenocysteine (SeC) (21). The human genome contains 25 selenoprotein genes, suggesting that Se plays an important role in the human body. Synthetic Se-containing compounds, including SeNPs and organic Se, have a wide range of biological functions, such as anti-oxidative and anti-tumor effects (22–24). These functions depend on the high level of intracellular reactive oxygen species caused by the high proliferation and metabolism of cancer cells, which increases sensitivity to Se oxidation damage. However, Se nanoparticles (SeNPs) have attracted considerable attention due to their optical, magnetic, and structural properties that molecules or bulk solids lack, as well as the low toxicity, faster absorption, and good biological activity (25, 26). Owing to their unique physicochemical properties, Se nanomaterials as drug carriers or therapeutic agents with good biocompatibility have demonstrated great potentials in the treatment of cancer in recent studies (27–31). SeNPs exert an anti-cancer effect at the nutritional level; however, high-level intake will convert it into an oxidant and lead to high toxicity. Interestingly, we found that lentinan-modified SeNPs have low toxicity, high permeability, and low blood clearance and could achieve efficient drug delivery to tumors and assist in the clinical treatment of solid tumors (32). However, determining the optimal nutritional concentration of SeNPs to effectively exert its anti-cancer effect is challenging. The present study investigated the anti-PCa effect of lentinan-functionalized SeNPs (LET-SeNPs) in combination with ZOL in the treatment of PCa.

## Materials and Methods

### Materials

Sodium selenite (Na_2_SeO_3_), chitosan (CS), polyvinylpyrrolidone (PVP), lentinan (LET), and vitamin C (Vc) were purchased from Guangzhou Chemical Reagent Factory (Guangzhou, China). Thiazolyl blue tetrazolium bromide (MTT), propidium iodide (PI), 4’,6-diamino-2-phenylindole (DAPI), 5,5’,6,6’-Tetrachloro-1,1’,3,3’-tetraacetyl carbocyanine iodide (JC-1), Lyso-tracker, Hoechst 33342, Annexin V-fluorescein isothiocyanate (Annexin V-FITC), vascular endothelial growth factor (VEGF), Matrigel, and Giemsa dye solution were purchased from Sigma–Aldrich (St. Louis, MO, USA). Ultra-pure water, purified by Millipore’s Milli-Q water purification system, was used in all experiments.

### Preparation of Different Modified SeNPs

Firstly, 1 mL of (10 mg/mL) CS, PVP, LET storage solution, and 1 mL of (10 mM) sodium selenite (Na_2_SeO_3_) storage solution were added to a glass bottle. Next, 1 mL of (40 mM) Vc and 2 mL of ultra-pure water were added. The mixture was stirred at room temperature overnight and dialyzed in Milli-Q ultra-pure water for 24 h. Subsequently, the concentration of Se was measured by inductively coupled plasma atomic emission spectroscopy (ICP-AES). This study used coumarin 6-labeled SeNPs to investigate the process of drug absorption by cells. The synthetic process was similar to that of SeNPs, adding coumarin 6 (20 μg/mL) prior to the addition of Vc.

### Structural Characterization of Different Modified SeNPs

The morphology and size of the nanoparticles were observed using transmission electron microscopy (TEM, Philips TECNAL-10). The hydrodynamic size and Zeta potential of different modified SeNPs were measured using a Malvern particle size analyzer. The energy dispersive spectroscopy (EDS, x-MaxTEM) element was analyzed by high-resolution TEM. The outer valence electron transition of the molecule was detected through the ultraviolet spectrum, and the skeleton structure of SeNPs was determined. The vibration of functional groups was detected by Fourier transform infrared spectroscopy.

### Stability of Different modified SeNPs Under Physiological Conditions

The stability and hemocompatibility of SeNPs were evaluated as previously described (33). Different modified SeNPs (0.5 mL) were incubated in phosphate-buffered saline (PBS), Dulbecco’s modified Eagle’s medium (DMEM), and human serum (0.5 mL). Changes in the hydrodynamic size of different modified SeNPs within 72 h were recorded using a Malvern nano-size analyzer (Zetasizer, Malvern Instruments, UK) to investigate their stability. To understand the biological safety of different modified SeNPs under physiological conditions, we simulated the changes in the physiological state of erythrocytes after administration. A volume of 0.5 mL of different modified SeNPs solutions (LET-SeNPs, CS-SeNPs, PVP-SeNPs; final concentration: 20 μM and 40 μM) and only ZOL solution (final concentration: 10 μM and 20 μM) were incubated with 0.5 mL of human erythrocyte suspension (centrifuged at 4°C and re-diluted with PBS) at 37°C for 4 h. A positive control group was formed using Triton × 100 (10 g Compact L, 0.5 mL) incubated with erythrocyte suspension (0.5 mL). Finally, the morphological changes in erythrocytes were observed using a light microscope (40×, Nikon ECLIPSE Ts2, Japan). At the same time, the supernatant of the mixture was added into a 96-well plate, and fluorescence was measured at 540 nm.

### Cell Culture and MTT Assay for Cell Viability

The prostate cancer cell line (PC3) used in this study was purchased from ATCC Global Biology Co., Ltd. (ATCC, Manassas, VA, USA). The cells were cultured in DMEM medium containing 10% fetal bovine serum and 1% double antibody (mixture of 50 U/mL streptomycin and 100 U/mL penicillin) and incubated under the following conditions: 37°C, 5% CO2, 95% relative humidity. Briefly, 2×10^4^ cells/mL PC3 cells were seeded into 96-plate. After 24 h, the cells were pretreated with SeNPs for 8 h, then cells were treated with different concentration ZOL for 72 h. Finally, the cell viability was determined by MTT assay. The synergistic effect of the drugs was analyzed using the equivalent line method (34, 35).

### Cellular Uptake and Localization of LET-SeNPs *In Vitro*

The uptake of green fluorescence-labeled LET-SeNPs by PC3 cells was measured and analyzed by flow cytometry assay (Beckman Coulter), while the subcellular localization was analyzed using a fluorescence microscope (Cytation 5, Bio Tek, USA). PC3 cells in the logarithmic growth phase were inoculated into 6-cm cell Petri dishes (cell density: 6×10^4^ cells/mL, 5 mL/dish). After allowing the cells to adhere to the wall for 24 h, the cells were incubated with coumarin 6-labeled LET-SeNPs (final concentration: 40 μM) for different treatment durations (0, 1, 2, 4, 6, 8, and 12 h), and the culture medium was discarded. Subsequently, the cells were washed with PBS and collected. Following horizontal centrifugation and re-suspension of the cells in PBS, the fluorescence value of the forward scatter area channel was detected using a flow cytometry assay (Beckman Coulter) and the absorption of the drug by PC3 cells was analyzed. Following treatment, the cells were incubated with Lyso-tracker (Red, 80 nM) for 2 h to label the intracellular lysosome, and treated with Hoechst 33342 (Blue, 1 mg/mL) for 20 min to label the nucleus. The lysosome was labeled with Lyso-tracker and showed red fluorescence, the nucleus was labeled with Hoechst 33342 and showed blue fluorescence, and the drug emitted green fluorescence. After staining, the medium was discarded, and the cells were rinsed thrice with pre-cooled PBS to remove any free drug and dyes. The fluorescence signals of cells were detected using a fluorescence microscope (Cytation 5, Bio Tek, USA), and the cell images with different fluorescence channels were captured.

### Cell Cycle Analysis

Cell cycle distribution was determined by flow cytometry (36). PC3 cells in the logarithmic growth phase were inoculated into 6-cm dishes (2×10^4^ cell/mL, 5 mL/dish) and cultured for 24 h until the cells adhered to the wall. A total of nine experimental groups were prepared as follows: blank control group, two different concentrations of ZOL (2.5 and 5 μM), two groups of LET-SeNPs alone (5 and 10 μM), two groups of LET-SeNPs (10 μM) combined with two different concentrations of ZOL (2.5 and 5 μM), and two groups of LET-SeNPs (5 μM) combined with two different concentrations of ZOL (2.5 and 5 μM). The experimental groups were pretreated with LET-SeNPs (final concentration: 5 and 10 μM) for 8 h, and the groups were cultured with different concentrations of ZOL (2.5, and 5 μM) for 72 h. After incubation, the cells were digested with 0.25% trypsin for 2–3 min, and the reaction was terminated by adding medium. Next, the cells were collected in a 15-mL centrifuge tube and centrifuged with washing solution (horizontal centrifuge: 1500 rpm, 10 min). Following centrifugation, the supernatant was discarded, pre-cooled 70% ethanol (1–2 mL) was added to each tube, and the tubes were placed in a refrigerator at −20°C overnight. The next day, the cell mixture was centrifuged (horizontal centrifuge: 1500 rpm, 10 min), washed with PBS, centrifuged in an Eppendorf tube (1.5 mL), resuspended in PBS, mixed with 300 μL of PI working solution (final concentration: 5 μM), and stained for 15 min at room temperature. Subsequently, the cells were washed and resuspended in PBS. The samples were analyzed using BD flow cytometry (≥10000 cells/sample), and the intracellular DNA content was detected using the FlowJo software (Phoenix Flow Systems, San Diego, CA, USA). The cell cycle at the Sub G0/G1, S, and G2/M phases, as well as the proportion of apoptotic peaks, were determined.

### Apoptosis Analysis

The effects of LET-SeNPs and ZOL on cell apoptosis were quantitatively analyzed by flow cytometry (37). PC3 cells in the logarithmic growth phase were inoculated into 6-cm dish (2×10^4^ cell/mL, 5 mL/dish) and cultured for 24 h until the cells adhered to the wall. Subsequently, the experimental groups were pretreated with LET-SeNPs (final concentration: 5 and 10 μM) for 8 h, and the groups were cultured with different concentrations of ZOL (2.5 and 5 μM) for 72 h. Following incubation, the cell culture medium was collected in a 15-mL centrifuge tube, and the cells were washed once with PBS. Next, the cells were digested with 0.25% trypsin solution (without ethylenediaminetetraacetic acid) for 2–3 min. The reaction was terminated with the addition of DMEM, followed by centrifugation of the collected culture medium, washing solution and cell mixture (horizontal centrifuge: 1500 rpm). Subsequently, the cells were washed once with PBS and centrifuged to remove the supernatant. The cells were re-mixed with binding buffer (100 μL), and mixed with Annexin V-FITC and PI staining solution (2.5 μL respectively). After thorough mixing, the cells were incubated at room temperature in the dark for 15 min. PBS (200 μL) was added to the flow tube cell suspension and flow cytometry assay (Beckman Coulter) was performed.

### Analysis of the Mitochondrial Membrane Potential

The decrease of the mitochondrial membrane potential is an important index which reflects cell apoptosis. When the mitochondrial membrane potential is high, the JC-1 dye accumulates in the matrix of the mitochondria to form polymers and produce red fluorescence. When the mitochondrial membrane potential is low, the JC-1 dye is a monomer producing green fluorescence. We measured the change of the mitochondrial potential based on the change of color and calculate the ratio of monomer to polymer to understand the cell damage caused by drugs. In the JC-1 membrane potential detection experiment, PC3 cells in the logarithmic growth phase were inoculated in 6-cm-cell dishes for 24 h (inoculation density: 1×10^5^ cells/mL; 5 mL/dish). The experimental groups were prepared as in the cell cycle experiment. The experimental groups were pretreated with LET-SeNPs (final concentration: 5 and 10 μM) for 8 h, and the groups were cultured with different concentrations of ZOL (2.5 and 5 μM) for 12 h. Subsequently, the culture medium was discarded, and the cells were digested with 0.25% trypsin. Next, the cells were washed twice with pre-cooled PBS and centrifuged to remove the supernatant (1500 rpm, 5 min); this was following by the addition of JC-1 dye (500 μL; working concentration: 10 μg/mL, PBS dilution) to an Eppendorf tube (1.5 mL) and incubation at 37°C for 30 min. The cell populations (JC-1 aggregation and JC-1 monomer states) were analyzed by flow cytometry assay (Beckman Coulter). The number of cells recorded in each experimental group was ≥10000.

### Cell Invasion

The matrix gel was dissolved at 4°C, diluted with fetal bovine serum-free medium, evenly added to the Transwell chamber (pore diameter: 8 μM, 30 μL/well), and placed into the incubator at 37°C for 4 h for solidification. PC3 cells in the logarithmic growth phase were added to the upper chamber covered with the matrix gel (serum-free medium mixed with 15×10^4^ cell/mL, 100 μL/well). Medium with 10% fetal bovine serum (500 μL) and 50 ng/mL VEGF were added in the lower chamber. The same concentration of drug was added in the upper and lower chambers, and the cells were incubated for 24 h. The following eight experimental groups were prepared: Control group; LET-SeNPs 5 μM alone group; two ZOL (2.5 and 5 μM) groups; and two LET-SeNPs 5 μM combined with different concentrations of ZOL (2.5 and 5 μM) groups. After 12 h of culture, cells in the upper chamber were fixed for 10 min with 4% paraformaldehyde. Subsequently, the fixative was removed, and the cells were stained with Giemsa stain and washed with ultra-pure water. Next, the non-invading cells and matrix gel in the chamber were gently removed using a cotton swab, and the cell invasion rate was observed and recorded under a microscope (Cytation 5, Bio Tek, USA).

### Cell Scratch Test

PC3 cells in the logarithmic growth phase were inoculated into 6-well plates (25×10^4^ cell/mL, 2 mL/well) for cell adhesion. When the cells reached almost 100% confluence, the combined experimental group was pretreated with LET-SeNPs (final concentration: 5 μM) for 8 h and Hoechst 33342 (50 ng/mL) stain was added for 15 min. Subsequently, the medium was removed, and scratches were made at the bottom of the vertical plate with a 200-μL pipette tip. The nonadherent cells were washed thrice with PBS. The cell images were captured using a fluorescence microscope (Cytation 5, Bio Tek, USA) and the state of cells was recorded. At the end of this step, ZOL at different concentrations (2.5 and 5 μM) diluted in serum-free medium containing VEGF (50 ng/mL) was added to each well. Next, the cells were cultured in an incubator. At 24 h and 48 h, the cell migration was observed and recorded using a fluorescence microscope (Cytation 5, Bio Tek, USA), and the scratch distance following migration was measured.

## Results

### Synthesis and Characterization of LET-SeNPs

**Figure 1:**
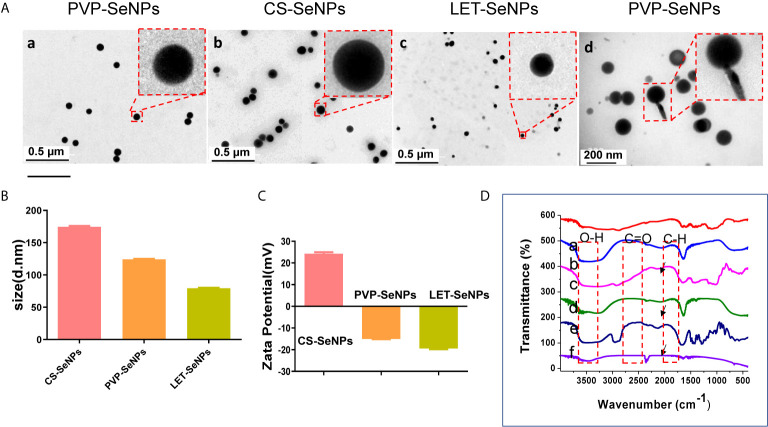
**(A)** Transmission electron micrographs of SeNPs modified by polyvinylpyrrolidone (PVP, a and d), chitosan (CS, b), and lentinan (LET, c). **(B)** Columnar chart of the particle size and **(C)** potential diagram of three different modified SeNPs. **(D)** Fourier transform infrared spectroscopy (FT-IR) diagram, and the dotted lines indicate CS, CS-SeNPs, LET-SeNPs, PVP, and PVP-SeNPs, respectively. The arrowhead denotes the position of the functional groups.

**Figure 2:**
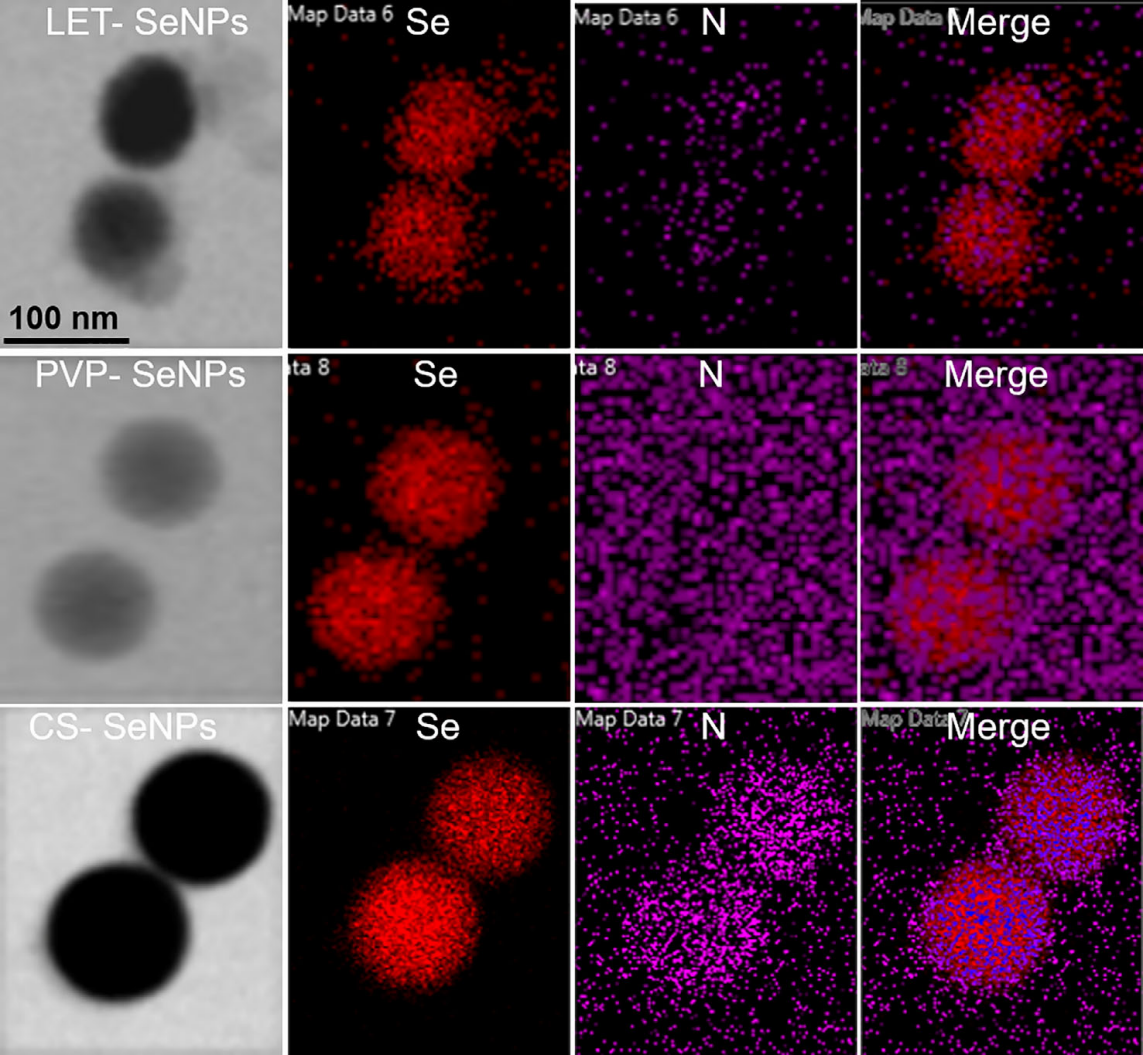
TEM-mapping analysis of CS-SeNPs, LET-SeNPs and PVP-SeNPs.

The LET-SeNPs have a spherical two-layer structure formed by Se atoms under the coating of lentinan. Lentinan is modified on the surface of the Se ball by intermolecular interaction. The morphology of the three types of modified SeNPs was observed by TEM. As shown in **Figure 1A**, the SeNPs modified by PVP, CS, and LET were spherical-like particles with different particle sizes. The TEM results showed that the particle size of CS-SeNPs was >150 nm, while that of PVP-SeNPs ranged between 100 and 150 nm. LET-SeNPs are colloids with a particle size <100 nm. As shown in **Figure 1Ac**. We analyzed the three types of SeNPs using the Zetasizer particle size analyzer (**Figure 1B**). We found that PVP-SeNPs and LET-SeNPs with relatively small particle sizes had a negative potential, whereas CS-SeNPs with a larger particle size were positively charged (**Figure 1C**). Fourier transform infrared spectroscopy (FT-IR) (as shown in **Figure 1D**) revealed that CS, LET and PVP have corresponding stretching oscillatory peaks around 3300 cm^-1^, 2350 cm^-1^, and 1385 cm^-1^, respectively. These characteristic absorption peaks are simultaneously shown in their respective infrared spectra of modified SeNPs. In addition, we also found the characteristic absorption peak of SeNPs at 1610 cm^-1^ on the infrared spectra of CS-, LET- and PVP-modified SeNPs. Moreover, as illustrated in **Figure 2**, the red Se atoms accumulated to form nanospheres. Also, the purple N element in the modified polymer was clearly visible, indicating the successful modification of various polysaccharides on the surface of SeNPs. The above results showed that the modification of SeNPs by polysaccharides was successful. Smaller particles size is more conducive to the cellular uptake of LET-SeNPs. Moreover, the sufficient negative charge of LET-SeNPs at -19.42 kept the nanosystems stable in complicated physiological environment. Hence, LET-SeNPs was more suitable than PVP-SeNPs and CS-SeNPs for cancer therapy.

### Enhancement of the Stability and Hemocompatibility of SeNPs

**Figure 3:**
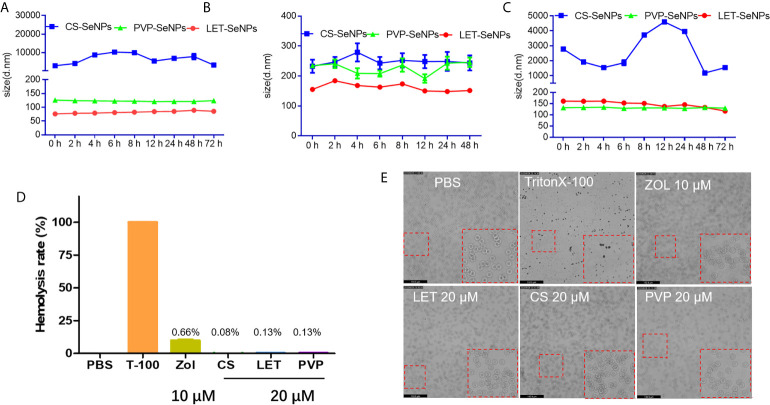
Stability and homocompatibility of different modified SeNPs and zoledronic acid (ZOL). Particle size change of polysaccharides modified SeNPs in **(A)** PBS, **(B)** DMEM (containing 10% FBS), and **(C)** HBS solution for 72 h. **(D)** Hemolysis of erythrocytes following treatment with polysaccharide modified SeNPs (concentration: 20 μM) and ZOL (concentration: 10 μM). **(E)** Morphology of red blood cells corresponding to the grouping of **(D)** Magnification, 20×.

Under physiological conditions, the stability of SeNPs modified by different polysaccharides is a very important aspect for the evaluation of their medical application (38). To investigate the stability of SeNPs modified by different polysaccharides, their size was measured in different solution environments using the dynamic light scattering method to evaluate changes in the hydration state. As shown in **Figures 3A–C**, the SeNPs modified by three different polysaccharides showed different degrees of stability in different solutions. LET-SeNPs and PVP-SeNPs were relatively stable in three different solution environments, whereas CS-SeNPs particle size exhibited greater instability in DMEM with 10% FBS. Among the three types of SeNPs modified by different polymers, the least change of particle size was the LET-SeNPs, followed by PVP-SeNPs, while CS-SeNPs were relatively unstable among the three solutions. Thus, LET-SeNPs have the best stability among the three types of SeNPs spheres, supporting its potential application in the field of medicine. The advantages offered by nanomedicines for biological application in the human body include a good biosafety profile and reliable *in vivo* circulation properties (39). However, under human physiological conditions, the hematocrit in blood reaches 37–45%. Thus, foreign NPs may cause some physiological stress reactions after entering the human body (40). Therefore, in this study, we tested the hemocompatibility of SeNPs functionalized with different polysaccharides (41). The hemolysis rates of ZOL and SeNPs with different surface modification were <5%, indicating their favorable safety profile (**Figures 3D, E**). For instance, the hemolysis of ZOL alone at the concentration of 10 μM was approximately 0.66%. The rate of hemolysis for CS-SeNPs, LET-SeNPs, and PVP-SeNPs was 0.08%, 0.13%, and 0.13%, respectively, without visible damage to the red blood cells. We observed that the red blood cells showed a smooth double concave disk shape. These results demonstrated that SeNPs have good blood compatibility.

### Cellular Uptake of LET-SeNPs

**Figure 4:**
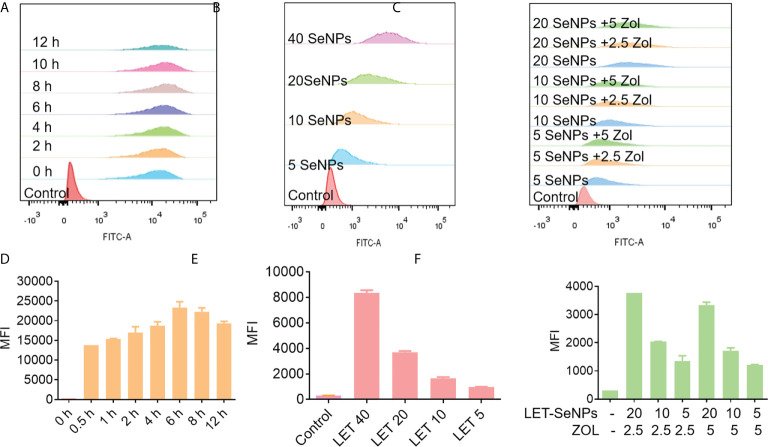
Cellular uptake of coumarin 6-labeled LET-SeNPs in PC3 cells analyzed by flow cytometry. **(A)** Cellular uptake of LET-SeNPs at different time points (0, 0.5, 1, 2, 4, 6, 8, and 12 h) at 40 μM. **(B)** Cellular uptake of coumarin 6-labeled LET-SeNPs at different concentrations (5, 10, 20, and 40 μM) for 6 h. **(C)** Cellular uptake of LET-SeNPs combined with ZOL at different concentrations for 6 h. Mean fluorescence intensity (MFI) of coumarin 6-labeled LET-SeNPs at **(D)** different time points, **(E)** different concentrations, and **(F)** different concentrations combined with ZOL.

Drug absorption and half-life time are very important indicators that affect the drug efficacy. Therefore, we used flow cytometric analysis to analyze the absorption effect of LET-SeNPs alone and in combination with ZOL. The absorption of coumarin 6-labeled LET-SeNPs increased gradually from 1 h to 6 h, reached the absorption peak at 6 h and, subsequently, showed a downward trend (**Figures 4A, D**). **Figures 4B, E** illustrate that the fluorescence intensity increased in parallel with the concentration, indicating that the absorption of LET- SeNPs is concentration-dependent. It was known that SeNPs is disassembled by lysozyme (42). We found that PCas internalized coumarin 6-labeled LET-SeNPs in a time dependent manner. However, the cellular uptake of coumarin 6-labeled LET-SeNPs showed a downward trend after 6 h, which may account for the reason that LET-SeNPs begins to metabolize into other products to exhibit its anticancer activities. According to **Figures 4C, F**, after the addition of ZOL (2.5 and 5 μM), the absorption of coumarin 6-labeled LET-SeNPs did not change significantly compared with that of LET-SeNPs alone but remained concentration-dependent. This result showed that addition of ZOL did not affect the absorption of SeNPs.

### Localization and Bio-response of LET-SeNPs

**Figure 5:**
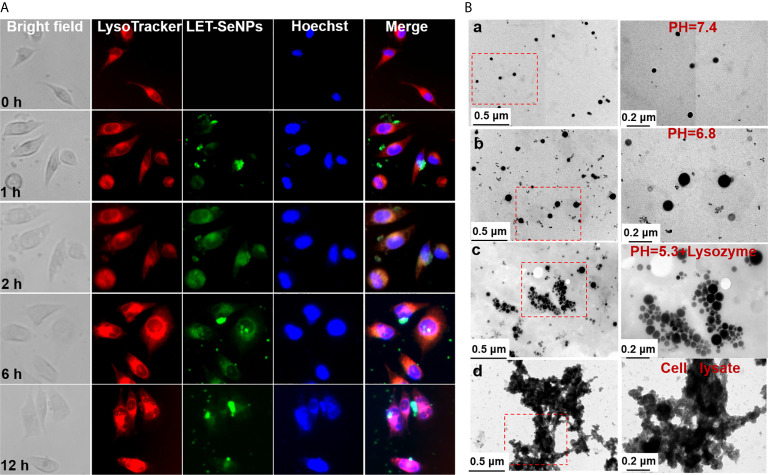
Localization of LET-SeNPs in PC3 cells. **(A)** Cellular localization of LET-SeNPs (10 μM) after incubation for different periods of time in PC3 cells. Nanoparticles (green), Lyso-tracker (red), and nucleus (blue). Scale: 50 μm; magnification: 400×. **(B)** TEM images of LET-SeNPs after incubation in different buffers (pH=7.4, a; pH=6.8, b; pH=5.3 + lysozyme, c; and cell lysate, d).

Next, we used fluorescence microscopy to examine the subcellular localization of LET-SeNPs after absorption. PC3 PCa cells were treated with coumarin 6-labeled LET-SeNPs and its distribution were observed at 0, 1, 2, 6, and 12 h. The red lysosome and blue nucleus were observed without drug absorption at 0 h (**Figure 5A**). From 1 h, the green SeNPs were gradually observed in the lysosome, and reached the peak at 4 and 6 h; moreover, the morphology of LET-SeNPs-filled lysosomes was clearly observed. The drug fluorescence decreased gradually at 12 h, indicating that the cell may have the drug metabolized. In the process of drug transport and metabolism, we observed the different morphological changes of SeNPs by TEM. There was no significant change in the particle size and appearance of LET-SeNPs in the simulated intravascular blood environment (pH=7.4) (**Figure 5Ba**). In the tumor microenvironment (pH=6.8), the particle size of LET-SeNPs expanded and the irregular edge of the particle sphere dissolved (**Figure 5Bb**). As the drug continued to be transported to the cellular lysosomal environment (pH=5.3) (**Figure 5Bc**), the NPs were dissolved into small particles of different sizes, and clusters and flocs were blurred with cellular proteins appearing after cell lysis and death (**Figure 5Bd**). In this metabolic process, SeNPs were relatively stable during transportation, and final degradation occurred only when they reached the tumor cells. These results suggest that, surface decoration with LET can enhance the stability and bio-response of SeNPs, and might reduce the development of undesirable side effects on human body.

### Synergistic Anti-Cancer Action of ZOL and SeNPs

**Figure 6:**
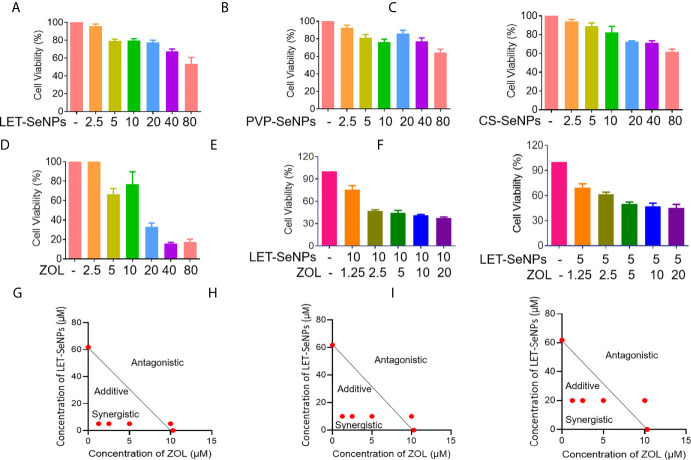
Synergistic anti-cancer activities of SeNPs and ZOL against prostate cancer PC3 cells. **(A–D)** Cytotoxic effects of LET-SeNPs, PVP-SeNPs, CS-SeNPs, and ZOL on PC3 cells for 24 h. **(E, F)** Synergistic effects of SeNPs with ZOL on PC3 cells. The cells were pretreated with ZOL for 8 h, then were treated with different concentration SeNPs for 24 h. **(G–I)** Isobologram analysis of the synergistic anti-proliferative effect of combined SeNPs (10, 5, and 20 μM, respectively) with ZOL on PC3 cells. The data points in the isobologram correspond to a 60% inhibition of growth in cells with combined treatment.

We used the MTT assay to evaluate the survival rate of cells treated with SeNPs and ZOL. Following treatment, the survival rate of PC3 cells decreased with the increase in the concentration of LET-SeNPs, PVP-SeNPs, and CS-SeNPs in a concentration-dependent manner (**Figures 6A–C**). The LET-SeNPs showed higher efficacy in killing cancer cells, most likely due to the anticancer activity of lentinan polysaccharide (43, 44). We also analyzed the synergistic effect using the equivalent line method. The results showed that, when IC60 was used, the concentration of LET-SeNPs alone and ZOL alone was 61.8 μM and 10.3 μM, respectively (**Figures 6D–F**). There was a synergistic effect of fixed concentration of LET-SeNPs at 10 μM with ZOL at 1.25, 2.5, and 5 μM (**Figures 6G–I**). Hence, LET-SeNPs at 5 μM in combination with ZOL was selected for further examination of the mechanisms of action.

### Induction of Cell Apoptosis and Cell Cycle Arrest at the S Phase by ZOL and SeNPs

**Figure 7:**
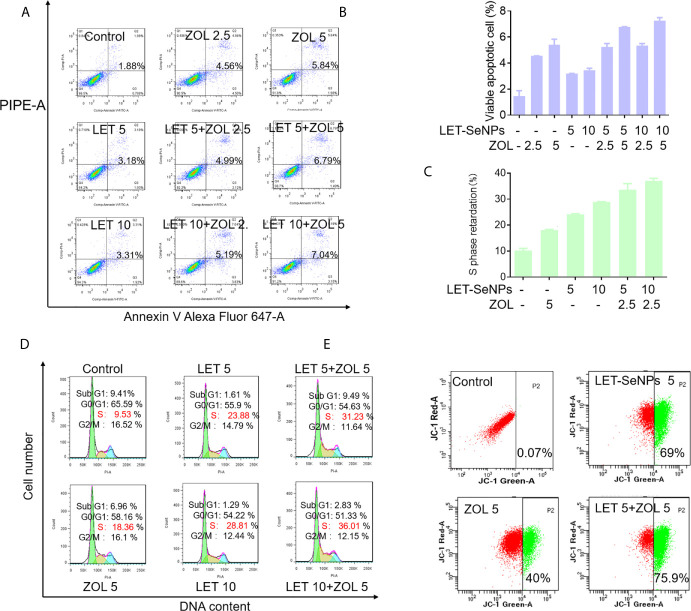
Induction of cell apoptosis and cell cycle arrest at S phase by ZOL and SeNPs. **(A, B)** Annexin V-PI co-staining assay to evaluate apoptosis in PC3 cells induced by LET-SeNPs (5 and 10 μM) and ZOL (2.5 and 5 μM). **(C, D)** Cell cycle distribution analysis of PC3 cells treated with LET-SeNPs (5 and 10 μM) and ZOL (5 μM). **(E)** Mitochondrial membrane potential in PC-3 cells following treatment with LET-SeNPs (5 μM) and ZOL (5 μM).

Further studies were performed to examine the anti-cancer modes of action of LET-SeNPs and ZOL in PC-3 cells. **Figures 7A, B** suggest that there is a certain dose-dependent increase of apoptosis in cells exposed to different concentrations of LET-SeNPs combined with ZOL. The apoptosis in the drug combination group was higher than that observed in the single drug control group. Specifically, the apoptotic cell of LET-SeNPs (10 μM) and ZOL (5 μM) was 7.04%. Thus, the co-treatment of LET-SeNPs and ZOL effectively induced PC-3 cells apoptosis. Furthermore, treatment with LET-SeNPs and ZOL either alone or in combination led to cell cycle arrest at the S phase, and the degree of cell cycle arrest increased in a drug concentration-dependent manner (**Figures 7C, D**). Correspondingly, the mitochondrial membrane potential also showed the same trend, and the proportion of green monomers representing early cell death gradually increased. The monomer ratio of individual ZOL 5 μM and LET-SeNPs 5 μM was 40% and 69%, respectively, and the combined use of drugs almost reached 76% (**Figure 7E**). These results suggest that, the combination of LET-SeNPs and ZOL may induce apoptosis and cell cycle arrest at the S phase. Firstly, LET-SeNPs with ZOL inhibited cell proliferation through S phase block, then co-treatment damaged mitochondria and subsequently caused mitochondrial membrane diffusion, and eventually induces apoptosis to achieve effectively cancer therapy.

### Inhibition of Cancer Cell Migration and Invasion by ZOL and SeNPs

**Figure 8:**
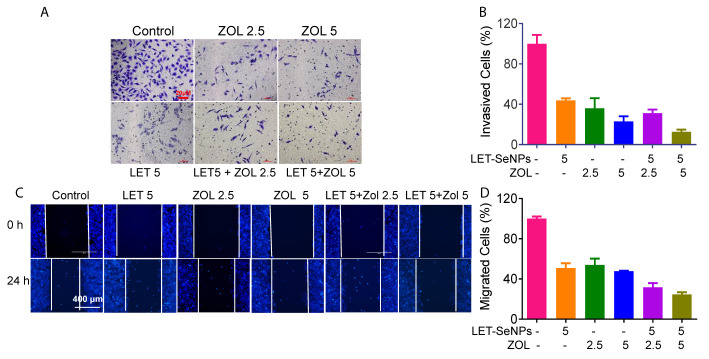
Inhibition of cancer cell invasion and migration of ZOL with SeNPs. **(A)** Wound healing assay and **(C)** anti-invasion effects of ZOL with LET-SeNPs on PC3 cells. The relative reduction in the width of the wound by the healing cells and the numbers of invaded cells suggested a remarkable anti-metastasis effect of combined treatment. **(B)** The migration cells in **(A, D)** invasion cells in **(C)** The quantitative data were analyzed by manual counting (% of control).

PCa cells are characterized by their ability to invade surrounding tissues. Moreover, they show a good cloning ability and can break through the basement membrane from carcinoma *in situ* to form metastatic foci and worsen the disease through the invasion-metastasis cascade reaction. We investigated whether the combination of ZOL and SeNPs could effectively inhibit the growth of tumor cells by inhibiting the migration and invasion of tumor cells (45, 46). According to the literature, ZOL in the concentration range of 1–100 μM is sufficient to inhibit the adhesion, migration, and bone resorption of PCa cells. The invasion experiment showed that the effect of the drug combination was significantly better than that of any single drug and positively correlated with the drug concentration (**Figures 8A, B**). ZOL alone (2.5 and 5 μM) exerted an inhibitory effect; however, this effect was more pronounced when ZOL was combined with LET-SeNPs. This effect was also reflected in the results of the cell migration experiments (**Figures 8C, D**). Therefore, ZOL and LET-SeNPs can effectively inhibitor the invasion and migration of PCa cells.

## Discussion

Combination chemotherapy has been postulated as a promising treatment strategy for cancer therapy. There are many advantages for combined treatment therapy, such as increasing drug efficiency and decreasing toxicity. A number of studies have demonstrated the benefit of chemotherapy strategy in combination with selenium, such as Selenocystine in combination with 5-fluorouracil, auranofin and doxorubicin, methylseleninic acid and cisplatin (47–50). Overall, the findings of this study improve the current understanding of the anti-tumor effect of Se. At present, there are numerous Se species available for anti-cancer applications, including inorganic Se, organic Se, and SeNPs. However, different types of Se species exert different biological effects due to their different morphology, chemical structure, specific metabolic pathways in cells and tissues, and variety of intracellular targeting molecules. SeNPs are nanoscale particles with unique advantages (e.g., low toxicity and fast absorption) compared with other Se (51, 52). The biocompatibility, bio-safety and excellent biological activities of Selenium particles have been confirmed to be superior than inorganic and organic selenium species, which indicates its potential application for cancer treatment. Additionally, lentinan has a lot of biological and physiological activities. Lentinan is a non-specific immune stimulant, which stimulates the proliferation of mononuclear macrophages and enhance the activity of T and NK cells. Additionally, the autoimmunity of patients with advanced prostate cancer is low. Therefore, the choice of LET-SeNPs may help to modulate the immune system of PCas and enhance the therapeutic efficiency ZOL. Further exploration and determination of the reference value are warranted for the clinical use of LET-SeNPs.

The results of our study showed that the anti-tumor effect of the combination of these two drugs was significantly enhanced. This is in consistent with the evidence regarding Se compounds previously reported in the literature. At present, the conventional chemotherapy drugs used in clinical practice are accompanied by severe gastrointestinal reactions, hair loss, and other side effects (41). Multi-cycle chemotherapy leads to a decline in patient compliance and affects the final treatment effect (42). The combination of drugs used in this study exerted a good anti-cancer effect, and can effectively overcome the issue of poor compliance. SeNPs are commercially available as a health product used to enhance immunity. However, when combined with ZOL (which acts through S-phase blockade), SeNPs can achieve the same or better anti-tumor effect. This simple dual-drug regimen can reduce the side effects caused by multiple drugs.

Evidences have shown that triggering ROS overproduction thereby activating p53 and MAPKs pathways to induce apoptosis is one of the important mechanisms of SeNPs in cancer therapy (53). Additionally, ROS overproduction triggering DNA damage and mitochondria malfunction thereby inducing apoptosis have also been confirmed to be the most important role of selenium for cancer therapy (54). Therefore, ROS-mediated signaling pathway may also contribute to the anticancer activities of LET-SeNPs and ZOL. We suggested that the killing of tumor cells by the combined drugs may be related to mitochondrial damage, and the imbalance between the BCL family and BCL family-related proteins. These effects induce the release of cytochrome C, leading to irreversible damage in cancer cells, DNA damage, nuclear fragmentation, decrease in cell size, and finally death.

Nevertheless, our study has limitations. This study is currently not supported by *in vivo* data, including animal and human experiments. In addition, there is no quantitative study on the specific mechanism of combined drug use.

Collective, this study revealed the good anti-tumor effect of the combination of LET-SeNPs and ZOL on PCa. The results will provide a rationale for the application of nanomedicine for the diagnosis and treatment of PCa.

## Data Availability Statement

The original contributions presented in the study are included in the article/supplementary material. Further inquiries can be directed to the corresponding author.

## Author Contributions

JZ: conception, design of research, edited, revised, and approved final version of manuscript. YA: performed experiments, analyzed data, prepared figures, drafted manuscript, and interpreted results of experiments. All authors contributed to the article and approved the submitted version.

## Funding

This work was supported by the Science and Technology Program of Guangzhou (202102010083), Guangdong Science and Technology Plan Project (2017ZC0005), the Guangdong Science and Technology Research Project of Traditional Chinese Medicine (20182020), and the Cultivation Program of the First Affiliated Hospital of Jinan University (802226).

## Conflict of Interest

The authors declare that the research was conducted in the absence of any commercial or financial relationships that could be construed as a potential conflict of interest.
